# Ultra-Light Airborne Measurement System for Investigation of Urban Boundary Layer Dynamics

**DOI:** 10.3390/s21092920

**Published:** 2021-04-21

**Authors:** Piotr Sekula, Miroslaw Zimnoch, Jakub Bartyzel, Anita Bokwa, Michal Kud, Jaroslaw Necki

**Affiliations:** 1 Faculty of Physics and Applied Computer Science, AGH-University of Science and Technology, 30-059 Krakow, Poland; zimnoch@agh.edu.pl (M.Z.); Jakub.Bartyzel@fis.agh.edu.pl (J.B.); Michal.Kud@fis.agh.edu.pl (M.K.); Jaroslaw.Necki@fis.agh.edu.pl (J.N.); 2 Institute of Meteorology and Water Management, National Research Institute, IMGW-PIB Branch of Krakow, 30-215 Krakow, Poland; 3 Institute of Geography and Spatial Management, Jagiellonian University, 30-387 Krakow, Poland; anita.bokwa@uj.edu.pl

**Keywords:** low-cost sensor, vertical profile, UAV, PBL, air pollution, response time

## Abstract

Winter smog episodes are a severe problem in many cities around the world. The following two mechanisms are responsible for influencing the level of pollutant concentrations: emission of pollutants from different sources and associated processes leading to formation of secondary aerosols in the atmosphere and meteorology, including advection, which is stimulated by horizontal wind, and convection, which depends on vertical air mass movements associated with boundary layer stability that are determined by vertical temperature and humidity gradients. The aim of the present study was to evaluate the performance of an unmanned aerial vehicle (UAV)-based measurement system developed for investigation of urban boundary layer dynamics. The evaluation was done by comparing the results of temperature, relative humidity, wind speed and particulate matter fraction with aerodynamic diameter below 10 μm (PM_10_) concentration vertical profiles obtained using this system with two reference meteorological stations: Jagiellonian University Campus (JUC) and radio transmission tower (RTCN), located in the urban area of Krakow city, Southern Poland. The secondary aim of the study was to optimize data processing algorithms improving the response time of UAV sensor measurements during the ascent and descent parts of the flight mission.

## 1. Introduction

Air quality in urban and suburban areas is a major long-term public health problem. Recent reports of air quality in Europe [1] indicate that many Polish cities do not meet the required standards. Many actions have been undertaken to improve the air quality in Poland. However, this is a complex problem that is connected to both the distribution and efficiency of pollution emission sources and local meteorological conditions. Particulate matter (PM) concentration is a parameter that is used to determine air quality. A high PM concentration affects human health [2,3], decreases visibility [4] and affects the global climate.

The concentration of individual compounds in ambient air depends on many factors, such as the location of emission sources, land forms, degree of urbanization and particular local environmental conditions. Studies of severe haze episodes in Beijing indicated that they are largely driven by meteorological conditions. High aerosol concentrations were recorded with weak wind conditions and air mass stagnation in the city [4]. The next step to better understand the dispersion of pollutants in urban areas is to investigate the boundary layer (BL) dynamics. A key element in such studies is to determine the vertical atmospheric profiles. Studies of vertical atmospheric profiles can be performed using devices such as balloon sounding [5], light detection and ranging (LIDAR) [6,7] and sound detection and ranging (SODAR) [8]. Selected studies indicate the possibility of a hyperfine vertical structure occurrence observed in the stable nocturnal boundary layer [9], which is related to the nonturbulent motions, microfronts or intermittent drainage flows. Another example of fine vertical structure was demonstrated by Pardyjak et al. [10], where a two-layer nocturnal structure was observed in a mountain valley. Cold pool occurrence and foehn air flow preventing the breakdown of stable boundary layer and trapping atmospheric pollutants close to the ground level in a mountain valley were presented in Li et al. 2015 [11].

Interest in air quality has significantly increased and inspired many companies to develop low-cost portable air quality sensors. In parallel, large progress in developing unmanned aerial vehicles (UAVs) is observed. These two factors recently enabled the use of sensors mounted on unmanned aerial vehicles for investigation of the boundary layer. Significant development of numerous low-cost PM sensors creates a new possibility of their use onboard a UAV to study vertical distribution of particles in many locations. It has been demonstrated that PM sensor models providing comparable results to reference sensors are Shinyei PPD42NS [12], Shinyei PPD60PV, Alphasense OPC-N2 [13], Plantower PMS1003 [14] and Plantower PMS3003 [15]. Long-term test results using a new model of Plantower PMS7003 conducted from 20 August 2017 to 24 December 2017 in Wrocław, Poland, show high correlations with the TEOM 1400a analyzer results [16]. Results of that study show good precision of this sensor because the variation coefficient was less than 7%. Tests using Plantower PMS7003 in indoor and roadside outdoor locations in Shanghai, China, also show good performance, with a high linear response and low bias values.

Aerosol distribution through the atmospheric column can provide valuable information about atmospheric compositions in specific layers [17,18,19].

Tests using UAVs to measure vertical profiles of the atmospheric parameters are becoming more common, and they are used to measure meteorological parameters, selected aerosols and gaseous pollutants, black carbon [20], methane, carbon dioxide [21] and ozone [22] concentrations. Using UAVs to study the lower troposphere provides new possibilities because UAV vehicles can cover large areas and monitor remote, dangerous or difficult-to-access locations, which increases operational flexibility and resolution over surface observations [23,24]. Comparison of results obtained using LIDAR and an optical particle counter (OPC) onboard a UAV [19] suggests that these two measurement are complementary methods for determining vertical aerosol concentration profiles with respect to altitude range characteristic for both methods.

Publications reporting the application of UAVs for measuring vertical atmosphere profiles in urban areas [25], along motorways [26,27] and in non-urban areas [23] have shown promising results. The first studies of the lower troposphere thermal structure over Krakow up to 3000 m a.g.l. were conducted in 1970–1972 using plane PZL 101 “Gawron” [28]. Research on the lower troposphere over the area of Krakow city was conducted using an airplane with a meteorograph on board and by using a weather balloon in 1978–1979. Measurements using sound detection and ranging (SODAR) equipment have been conducted since 1980 by the Remote Sensing Department of the Institute of Meteorology and Water Management, National Research Institute (IMWM-NRI), in Krakow-Czyzyny. Measurements using SODAR showed that a 24-h ground inversion or elevated inversion may occur in Krakow on up to 20% of the days each year. A higher upward surface long-wave radiation flux and lower atmospheric dynamics during the cold periods cause the 24-h inversion. Based on the SODAR data, 86% of cases had stable atmospheric conditions at night and were associated with temperature inversions during the winter. During the remaining 14%, conditions were neutral and unstable. Detailed information about diurnal and annual variability of the thermal stratification in Krakow based on SODAR SAMOC-4c measurements from 1994–1999 was presented by Godłowska [29].

The aim of this paper was to demonstrate an airborne system for performing measurements of meteorological (temperature, relative humidity, atmospheric pressure and wind speed) and air quality parameters (PM_10_) inside the boundary layer up to a few hundred meters above ground level based on a low-cost sensor set installed on board of a UAV. The article presents configuration of the system, calibration of the sensors and full sequence of a data postprocessing procedure consisting of data smoothing and correction of sensors’ response time. Finally, the performance of the system is demonstrated in the example of three atmospheric profiling measurement campaigns by comparing obtained results with other available static measurement points available in the vicinity of flight location. According to our literature studies, this is one of the few articles presenting a complete and comprehensive description of the full data flow algorithm validated by the analysis of real cases [21,30,31]. The system has been developed by the Environmental Physics Group at the AGH University of Science and Technology.

The second section presents the architecture of the system, sensor calibration and numerical procedures aimed at improving data quality consisting of time response error correction and smoothing procedure. Section Results presents the performance of the system demonstrated by the example of three measurement campaigns performed in Krakow, Southern Poland. These examples were aimed at the characterization of the transition phase of the boundary layer between stable and convective after sunrise and convective and stable after sunset. The presented results allowed us to determine the impact of the formation of convective and stable nocturnal boundary layers during the transition phase on vertical pollutant distribution in urban areas. To date, only a few studies presenting the formation of convective and stable nocturnal boundary layers using a drone measurement system in the city have been reported, e.g., [32].

## 2. Materials and Methods

### 2.1. AirDust Measurement System

**Figure 1:**
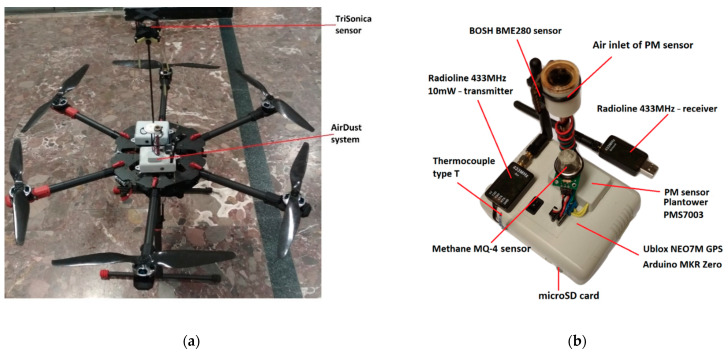
Unmanned aerial vehicle (UAV) equipped with air quality and meteorological components sensors (**a**) and AirDust ultra-light measurement system (**b**) for temperature, relative humidity, atmospheric pressure, particulate matter and GPS position equipped with a data logger.

Figure 1a presents an unmanned aerial vehicle (UAV) equipped with air quality and meteorological component sensors. The ultra-light measurement system dedicated for temperature, relative humidity, pressure and particulate matter (PM_10_ fraction) measurements is presented in Figure 1b. It was built based on the Arduino MKR ZERO microcontroller, which is responsible for communication with the sensors, storing the measurements on the memory card and sending information in real time to the ground station using a 433-MHz radio line.

The system was powered by a drone battery, and for this purpose, a step-down converter reducing the input voltage from ca. 22 V to 5 V at 2 A was used. Temperature, relative humidity and atmospheric pressure were measured using a digital humidity, pressure and temperature sensor (BME280, Bosch Sensortec GmbH, Reutlingen, Germany). It communicated with the microcontroller via an I2C interface. Additionally, the measurement system was equipped with a thermocouple type T to monitor rapid temperature changes. For PM_10_ measurements, a PMS7003 low-cost sensor (Plantower, Beijing, China) based on the light scattering method was used. To reduce the impact of water vapor condensation at high humidity and low air temperature on PM measurement, sensor air inlet was heated. Additionally, to reduce the air turbulences at the air inflow to the sensor, the air inlet was equipped with a cap located in the upper part of the drone providing a laminar flow during the drone’s climb. The measurement system was supplemented with the NEO-7 GNSS module (u-blox AG, Thalwil, Switzerland) allowing monitoring of the horizontal and vertical sensor positions. Additionally, the system is equipped with an electrochemical methane (CH_4_) sensor for detection of high concentration natural gas plumes, but this part was not used in the presented study. A list of components and their technical parameters provided by a producer is included in Figure 1 and Table 1. The whole system is housed in 11 × 9 × 5 cm 235 g of weight box fixed to the drone platform.

### 2.2. TriSonica Wind Sensor

**Figure 2:**
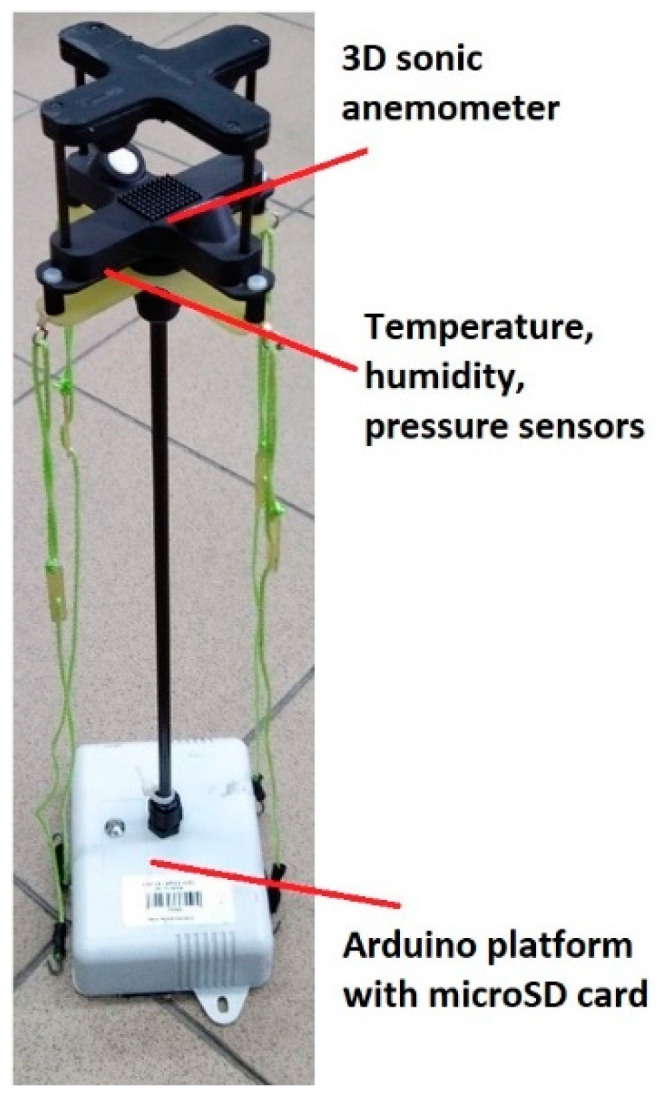
TriSonica^TM^-based anemometer system.

The second element of the presented airborne measurement system is an acoustic wind sensor using a TriSonica^TM^ Mini Wind and Weather Sensor (Anemoment LLC, Longmont, CA, USA). This module is also equipped with a data logging module based on the Arduino MKRZERO microcontroller and the NEO-7 GNSS module (u-blox AG, Thalwil, Switzerland) allowing monitoring of the horizontal and vertical sensor positions. Wind sensor is a 50 g miniature 3D sonic anemometer supplemented with temperature, pressure and humidity sensors (located at the bottom side of the TriSonica sensor), magnetometer and 3D accelerometer (Figure 2).

The anemometer measures wind speed (0–50 m·s^−1^) and wind direction (0–360°) along with the actual position of the sensor with a 10 Hz data rate. To avoid measurement disturbance due to turbulences generated by the drone’s propellers, a wind sensor is fixed on a small boom ca. 30 cm above propellers’ level (see Figure 2). The idea of wind direction correction using an onboard magnetometer during rotation of the drone during the flight was tested. The results show that magnetic field disturbance generated by the currents supplying the drone’s motors makes it impossible to use this sensor for a correction procedure. Reliable wind direction data can be acquired only by avoiding drone rotation during the flight. A list of components and their technical parameters is included in Table 2. Test of air temperature, air humidity and atmospheric pressure sensors in the TriSonica sonic anemometer showed that their inertia is more significant than sensors used in the AirDust system (not shown in the article). Sensors are located inside the anemometer shield which causes their poor ventilation and longer time response. Due to this fact, only measurements of wind components and sonic temperature were used in the analysis.

### 2.3. Sensors Calibration

Calibration of meteorological and wind sensors was performed by comparison with the stationary meteorological station at the Faculty of Physics and Applied Computer Science, AGH University of Science and Technology (AGH UST). The meteorological station was located on the roof of the faculty building. Air temperature, relative humidity and atmospheric pressure, at the level of 20 m a.g.l., were measured using a VAISALA WXT520 platform. Additionally, particulate matter fraction measurements (PM_10_) were conducted at the same point by a TSI 8530 optical aerosol monitor calibrated using the gravimetric method. 

**Figure 3:**
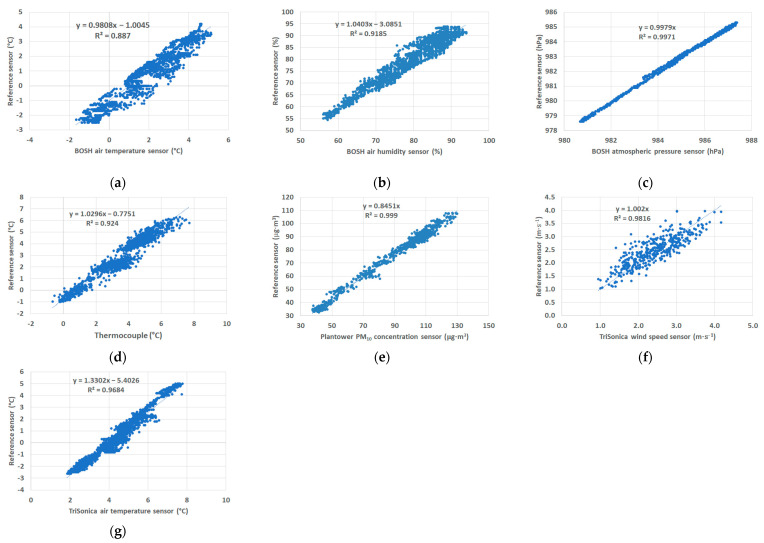
Comparison of the tested sensors with reference instruments: (**a**) air temperature—BOSH sensor; (**b**) air humidity—BOSH sensor; (**c**) atmospheric pressure—BOSH sensor; (**d**) air temperature—thermocouple; (**e**) PM_10_ concentration—Plantower sensor; (**f**) TriSonica^TM^ wind sensor, (**g**) TriSonica^TM^ sonic temperature.

Figure 3 presents the comparison of tested sensors with reference instruments for three days of tests with one-minute resolution (VAISALA WXT520 platform saves data once per minute). The calibration equation and correlation coefficient R^2^ were obtained using linear regression. In the plots in Figure 3, there are presented linear regression equations for individually tested parameters. For calibration of the BOSH atmospheric pressure and PM_10_ concentration sensor, the intersection points of the straight lines matching the graph were set to 0. Tests of the Plantower sensor showed the correct measurement for a concentration close to 0 μg⋅m^−3^. Correlation coefficient R^2^ calculated for all sensors except the BOSH air temperature sensor was higher than 0.9. The highest correlation coefficient value was obtained for the PM_10_ concentration sensor and the BOSH air humidity sensor (above 0.99). Calibration of sensor Plantower indicated that concentration of PM_10_ was overestimated, therefore the slope of a regression line is equal to 0.85. Accuracy of all sensors used in the study calculated based on the accuracy provided by the manufacturer and calibration were equal for: temperature sensors from 0.6 °C (thermocouple) and 0.7 °C (BOSH temperature sensor) to 1.2 °C (TriSonica^TM^—sonic temperature) in the range from 0 to 20 °C; for BOSH air pressure sensor, it was equal to 0.6 hPa (in the range of 300–1100 hPa, 0–65 °C); 2% for BOSH air humidity sensor (in the range of 20–90%); 0.1 m⋅s^−1^ for TriSonica^TM^ wind speed sensor (range from 0 to 10 m s^−1^); 6 μg⋅m^−3^ for PM10 sensor error in the range from 0 to 100 μg⋅m^−3^.

Formulas of linear regression were used to correct the systematic error of measurements presented in this article.

### 2.4. Smoothing Procedure

**Figure 4:**
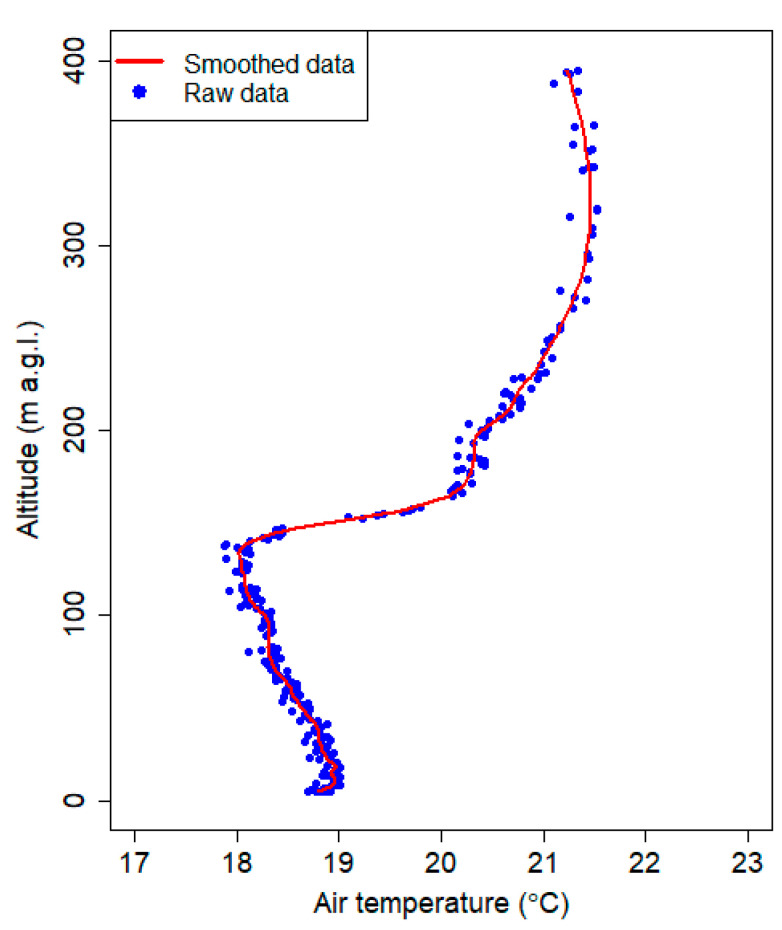
Smoothing data using local polynomial regression and the vertical profile of the temperature measured on 3 August 2018 at 3:23 UTC.

To remove the noise related to the sensors, the local polynomial regression fitting method was used. Locally estimated scatterplot smoothing (LOESS) is one of the most common representations of the above procedure that combines multiple regression models in a k-nearest-neighbor-based meta-model [33]. At each point within the data set range, a low-degree polynomial was fitted to a data subset. The polynomial was fitted using weighted least squares, giving more weight to the points near the area where the response was being estimated and less weight to points that were further away. The value of the regression function for the point was then obtained by evaluating the local polynomial using the explanatory variable values for that data point. The LOESS fit was complete after the regression function values had been computed for each from the n data points. The method was implemented using R software, and it was called the LOESS function [34]. The vertical profile of air temperature without smoothing (raw measured data) and the results of the LOESS method are presented in Figure 4. The degree of smoothing was set to 0.1, which means that 10% of the data were used in the calculations for each point. Using higher values caused data smoothing that was too strong, and therefore, too much valuable information about the atmospheric vertical profiles was lost. The degree of the polynomial used in the calculation is a default value that was equal to 2. The smoothing method was used to remove noise from the vertical profiles of the air temperature, air humidity, air pressure and PM_10_ concentration profile.

### 2.5. Correction of Sensors’ Response Time

Calibration of sensors presented in Figure 2 was made for slow-changing meteorological parameters, therefore inertia of the tested sensor was invisible. During the evening or early morning, measurements of vertical profiles of atmosphere differences between ground measurements and measurements at a few hundred meters can be significant. Therefore, measurements of meteorological components [35] and gas concentrations [36] in the atmosphere are subject to several sensor-related sources of error. One of the most common in atmospheric measurements is time-lag error, which occurs in radiosonde profiles [37] or mobile measurements [38]. It is caused by the fact that sensors need a certain time in order to reach equilibrium with their ambient environment. To correct this error for humidity and temperature sensor, the procedure based on Equation (1) [39] time-dependent response presented below was developed.

(1) $$\frac{dU_{m}}{dt}=\frac{1}{k}\cdot \left(U_{a}-U_{m}\right),$$

*U_a_* and *U_m_* are ambient and measured parameter values, respectively; the coefficient *k* is sensor time constant dependent on atmospheric conditions. “The sensor time constant is not the same for all atmospheric conditions. For temperature sensors, the sensor time constant is found to be primarily dependent on the atmospheric pressure (air density) and the ventilation of the sensor. For relative humidity sensors, the sensor time constant is additionally a function of absolute temperature and water vapor content. A decrease in temperature and atmospheric pressure leads to increased sensor response times, and so does a lack of ventilation and low water vapor content” [39]. Time-lag error in vertical profiling depends also on the vertical velocity of sensors.

Solving Equation (1) for *U_a_* provides a simple way to compute a true ambient parameter from measurements [36]. Before calculation of a derivative, original datasets had to be smoothed. Earlier studies of this issue confirmed the need to smooth the data before correction [37,38,39]. To compute derivative *dU_m_*/*dt*, a 4th-order Savitzky–Golay filter was used [40,41] with a length of 11 samples, without losing detailed information of the vertical structure of the atmosphere. Calculated derivative *dU_m_*/*dt* was smoothed to omit sudden changes (peak values). It should be noted that to compute a derivative, homogenous-in-time data with known time resolution are needed.

To remove the noise related to the sensors, the local polynomial regression method was used, and detailed information about this method is presented in Section 2.3.

The coefficient k was determined for each flight by an optimization that minimizes the difference between the corrected humidity and temperature profiles for ascent and descent profiles.

**Figure 5:**
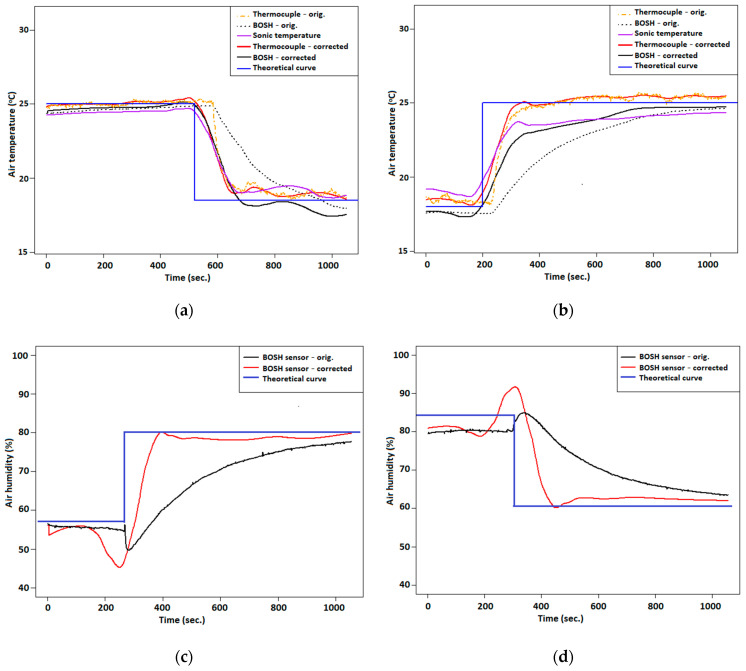

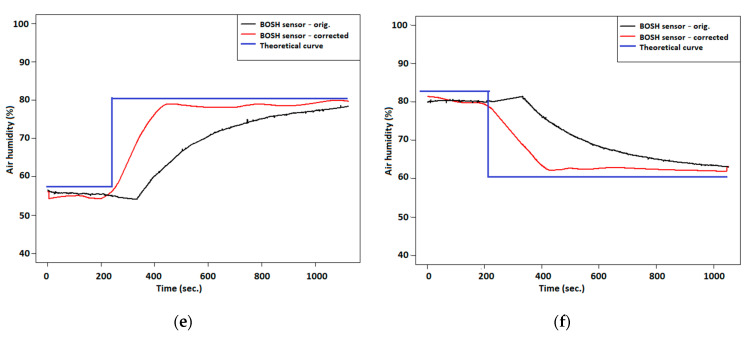
Test of response time correction in the outdoor-indoor conditions test: (**a**) temperature sensors response—cooling; (**b**) temperature sensors response—heating; (**c**) air humidity sensor response—moistening; (**d**) air humidity sensor response—drying; (**e**) air humidity sensor response—moistening (with data overshoot correction); (**f**) air humidity sensor response—drying (with data overshoot correction).

In order to validate the calculations, tests of time response of temperature and humidity sensors for big amplitudes of temperature and humidity in indoor-outdoor tests in the cold season were conducted. For this case, the sensors were moved inside and outside the window assuming that the time course of temperature and humidity should be a quadratic wave. By comparing results with the theoretical time course, k coefficient was calculated. Tests indoor-outdoor lasted several minutes until sensors stabilized. Obtained time curves of temperature and humidity were smoothed using 10% of the data. Calculation of derivative *dU_m_*/*dt* was obtained by using 11 points. Calculated derivatives’ curves were also smoothed by using 10% of data. Figure 5 presents an example plot of air humidity and temperature time series before and after correction. At the moment of putting outdoor or indoor humidity sensor, a sudden peak of humidity was observed (caused by condensation and drying of the plate on which the sensor is located)—Figure 5e,f. In order to omit enhancement of the sudden peak of humidity observed during the change of air conditions, some data were removed, and results are presented in Figure 5c,d. Optimal k coefficient was found to equal 20, 120 and 220 for the thermocouple, BOSH temperature sensor and BOSH humidity sensor, respectively.

Tests of temperature change rate at indoor-outdoor conditions pointed out that time response and temperature change rate of the thermocouple was significantly faster than the BOSH sensor. After applying a correction method to the thermocouple and BOSH time course of temperature, differences between both sensors’ responses were smaller.

Detailed information about the time response of the sensor, length of sensor stabilization and temperature/humidity amplitude are included in Appendix A Table A1. Analysis of results presented in Figure 5 pointed out that the time response of temperature sensors on condition change was faster even by 60 s; before correction of the time course of the thermocouple, there was a visible sharp change of temperature in the first few seconds. Due to the fact that the optimal k coefficient for the thermocouple is relatively small compared with the coefficient for the BOSH temperature sensor, the rate change of temperature in 1 s is higher from the reference value by 15%–20%. Significant differences between reference time course and time course after correction are visible at air temperature and humidity courses from the BOSH sensor (Figure 5a–d). Length of the period during temperature or humidity change in the range from 10% to 90% of maximum amplitude is two or even three times shorter than the reference value.

However, differences of the rate of parameter change in time after correction are visible between heating and cooling of the sensors, from 15–20% lower for the thermocouple and air humidity BOSH sensor to 140% lower for temperature BOSH sensor for heating.

Presented procedures were applied to vertical profile correction. During the flight operation, vertical drone speed can differ slightly, among others during landing and take-off or caused by the disturbances (wind gusts), and due to this fact density of points in the vertical profile slightly fluctuates. Because of uneven distribution of measuring points as a function of height, smoothing original data and calculated derivative were made to the altitude, not time. Smoothing procedure was applied to the original data and calculated derivative. Depending on the amount of measurement points, the length of the sample used for smoothing varied from 20% to 30% of all data in the profile.

The flight corresponding to the ascent and descent of the drone was treated as two separate sets of data; for one data set containing both phases, the method did not give promising results.

In order to determine the convergence of the vertical profile measured during the ascent and descent parts of the profile, the mean square error (RMSE) value was determined. Calculations of RMSE were conducted for the pair of points with an altitude difference of less than 4 m.

Because during the last phase of landing at a low altitude disturbances generated by drone are significant, measurements at the lowest layer up to altitude 10– 15 m above the ground level were omitted.

**Figure 6:**
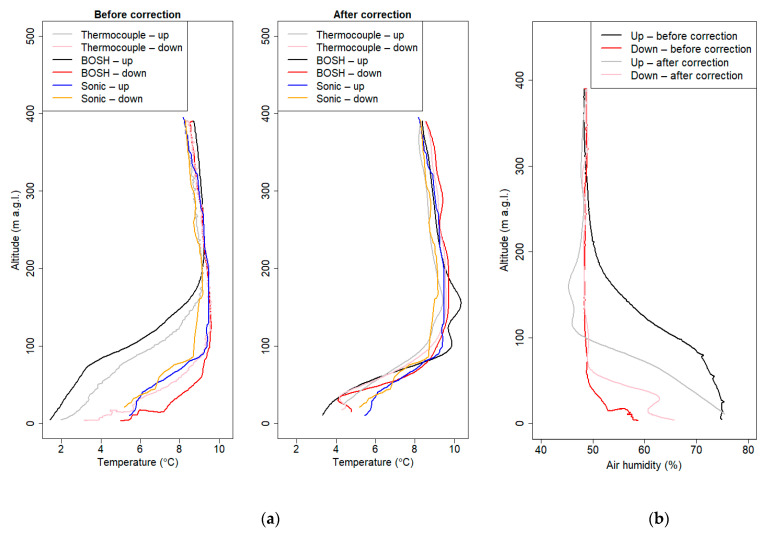
Vertical profiles of the atmosphere before and after correction method—flight at 7.3.2019 6:11 UTC: (**a**) air temperature; (**b**) air humidity.

Figure 6 presents the examples of temperature and humidity vertical profiles before and after correction.

Coefficient k for the thermocouple and temperature BOSH sensor was set to 24 and 44, respectively. Optimal coefficient for the BOSH air humidity sensor was found to be 50. After the correction method, we could observe that differences between ascent and descent vertical profiles were significantly smaller. Differences between the BOSH sensor and thermocouple at an altitude between 0 and 200 a.g.l. were reduced by the presented procedure. Air humidity profiles during descent and ascent of the drone after correction procedure still differed at the lowest part of the atmosphere, but a significant improvement was observed. Relative humidity profiles presented in Figure 6b show that this method has still some limitations.

### 2.6. Study Area

**Figure 7:**
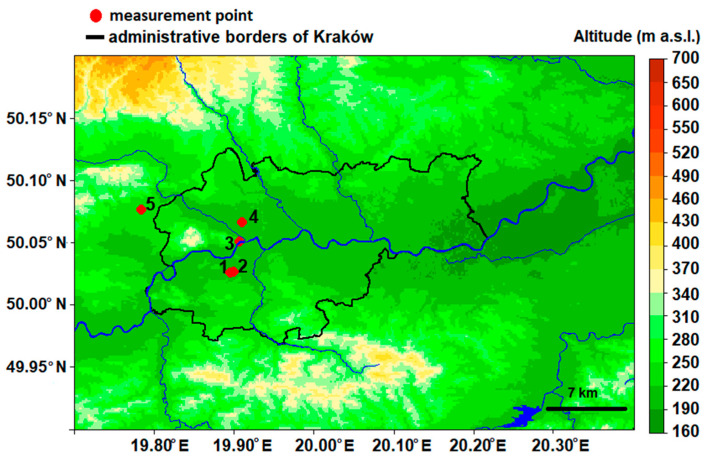
Location of measurement points in Krakow.

Krakow is the second-largest city in Poland (area: 327 km^2^, population: 771,000 inhabitants), located in the Lesser Poland region, in a large valley of the Vistula River. The city’s area includes three different regions and geological structures, i.e., the Polish Uplands, the Western Carpathians and the basins of the Carpathian Foredeep [42]. The dominant part of the city is located in the third region, with a diversified natural environment. The hilltops bordering the city to the north and the south reach about 100 m above the river valley floor, similar to the hilltops in the western part of the valley which means that the city is located in a semi-concave land form (open only to the east) and sheltered from the prevailing western winds (Figure 7). This location is responsible for frequent air temperature inversions and the prevailing weak western winds, which contribute to the poor ventilation in the city. A clear reduction in the wind speed in the city is reflected by the frequent incidence of atmospheric calm. Analysis of data from 2001–2010 indicated that there was atmospheric calm in Krakow during an average of 16.8% of the year [43]. Mobile measurements of air temperature during calm and cloudless nights indicate that most probably katabatic flows occur on the slopes of the hills which surround the city, but the resulting cold air pool is formed only in rural areas, i.e., those flows do not enter the urbanized area and have no impact on the urban canopy layer inside the city [44].

Vertical profile measurement data were collected on the Jagiellonian University (JU) Campus (UAV place; Figure 7). The flight point was located in the western part of the city; the terrain was covered with grass, and altitude differences of the surrounding area were up to 15 m (average value, 209 m a.s.l.). The flights were performed up to a height of 500 m a.g.l., and the maximum flight height was determined by the limitations of the aircraft. The location of the vertical measurements using a UAV was chosen based on safety requirements (e.g., distance from buildings and people), and it was a location that was close to the city center that was representative for incoming air masses.

Vertical profile measurements representing the lower part of the profile (altitude up to 5 m a.g.l.) were compared with the observations from a meteorological station located at a distance of 300 m east from the flight point on the JU Campus (Figure 7). Small differences between measurement results that originated from flights and the meteorological station may be caused by the influence of microclimatic conditions in both locations. The flight point is located in open space, while the station is located between three-story University buildings.

Vertical profile measurements of air temperature and relative humidity corresponding to altitudes of 50 m a.g.l. and 100 m a.g.l. were compared with the measurements from the RTCN tower, located 2.7 km north from the flight point. The location of the RTCN tower is shown in Figure 7. Temporal resolution of meteorological parameters from the JU Campus and RTCN tower stations was equal to 10 min; for the AGH UST station, temporal resolution was equal to 1 min; measurements from the synoptic station of Balice had hourly resolution. Location of measurement points used in the study is included in Table 3.

Explanations: numbers as in Table 3. Topographic data used in Figure 7 come from Shuttle Radar Topography Mission database provided by the National Aeronautics and Space Administration (https://www2.jpl.nasa.gov/srtm/ (accessed on 18 April 2021)).

### 2.7. Measurement Periods

From December 2017 to November 2018, more than 60 vertical profile test measurements of the atmospheric parameters were performed in Krakow. In the present study, measurements taken on the following 2 days (12 flights) were analyzed:

- 18 September 2018 from 15:00 UTC to 21:00 UTC, i.e., afternoon hours;
- 21 September 2018 from 4:00 UTC to 9:30 UTC, i.e., morning hours.

The aim of the measurements was to study the dynamics of the atmospheric boundary layer during transition phases between the convective boundary layer (CBL) and stable boundary layer (SBL) after sunset and between SBL and CBL after sunrise. The measurements were made during weather conditions controlled by a high-pressure system, with clear sky and weak wind at the ground level. High-pressure weather in the summer season is favorable for fog formation and a strong thermal inversion at night and strong convective mixing during the day.

In 2019, the drone system was expanded by a TriSonica sonic anemometer sensor. An example of wind profiles associated with other measured parameters in profiles is presented for the flight campaign performed in March 2019.

Observations during the measurements, synoptic comments (available at http://www.meteo.pl/ (accessed on 18 April 2021)) and surface pressure charts (available at https://danepubliczne.imgw.pl (accessed on 18 April 2021)) were used to describe the meteorological conditions during the measurement campaigns.

### 2.8. Vertical Profile Measurements

Each measurement campaign consisted of several vertical flights performed over the same location approximately every hour. The duration of each flight lasted from 5 to 15 min depending on the maximum altitude and the climb velocity, which ranged from 1 m s^−1^ to 2 m s^−1^. The maximum drone climb velocity (2 m s^−1^) was determined based on the assumed flight altitude and battery operating time. It was tested that, for velocities below 2 m s^−1^, the obtained results after applying all postprocessing algorithms described in Section 2 reflected actual variability in the vertical profile. Because of strong turbulence generated by the multirotor propellers below the aircraft, the sensors were located on the upper part of the drone, and only the ascending part of the flight was analyzed.

## 3. Results

In this section, vertical profile measurements from three campaign days are analyzed. Figures presented in Section 3.1, Section 3.2, Section 3.3 show vertical profiles of the air potential temperature, relative humidity and PM_10_ concentration for all campaigns. Additionally, for the third campaign wind speed profiles are presented in Section 3.3. In subsections below are presented the time series of air temperature, humidity and wind speed recorded at the surface station of the JU Campus. The red points added to charts represent measurements conducted by the UAV platform at the start of each flight. Thermal stratification of the atmosphere in Krakow during the measurement days are presented in the last Figures at Section 3.1, Section 3.2, Section 3.3, using data from two different levels at the RTCN tower (50 and 100 m a.g.l.). Points presented in those Figures represent measurements of air temperature made by a drone during each flight at the altitudes that were closest to the tower sensors’ elevations.

Due to the fact that the measurement campaign was conducted during nighttime and daytime conditions, temporal variation of vertical PBL structure was observed. During each campaign period, ground thermal inversion with and ground layer with increased air humidity were observed. Depending on temporary conditions, the depth of both layers could be different.

**Figure 8:**
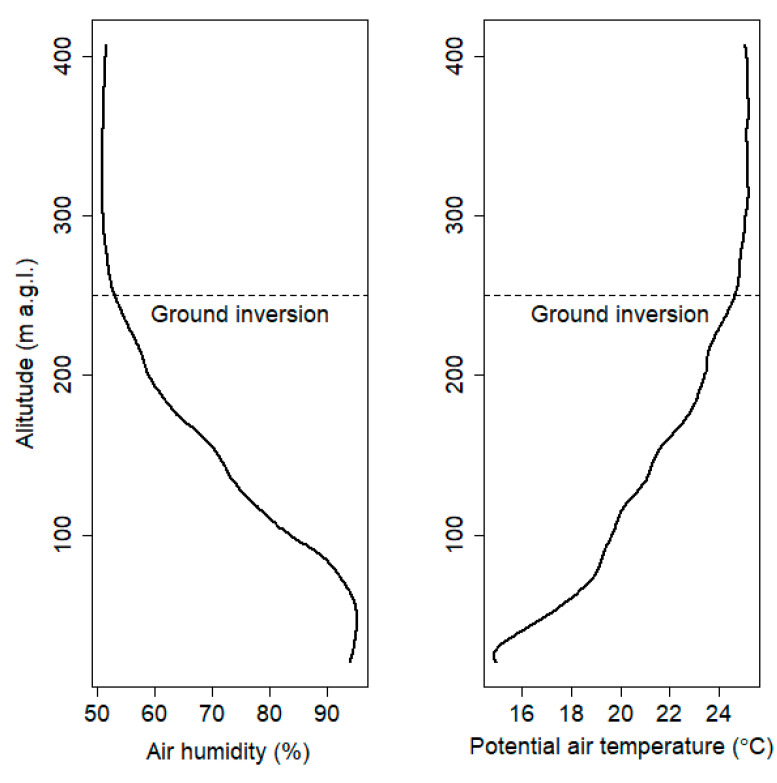
Ground inversion layer height at sixth flight (18 September 2018).

For this purpose, the vertical gradient of temperature/humidity was calculated. A location where significant gradient change is observed in the vertical profile of potential temperature is assigned as estimated ground inversion layer height (see Figure 8). Studies of the vertical air humidity profile and its vertical stratification will be continued in further work in order to better understand the phenomenon. Atmospheric stability was estimated based on the vertical gradient of potential temperature [45]. Based on the analysis of all vertical profiles from the measurement campaign of potential air temperature and climatological analysis of the analyzed region (not shown) for neutral and stable conditions, potential temperature gradient interval was set to ± 0.7 °C/100 m and >0.7 °C/100 m, respectively. Cases of unstable atmospheric conditions with the potential gradient lower than −0.7 °C/100 m were not observed. General information presenting characteristics of relative air humidity and potential air temperature vertical profiles are included in Appendix A, Table A2, Table A3 and Table A4.

### 3.1. 18 September 2018—Evening Campaign

Between 17 and 21 September 2018, Poland was under the influence of a high-pressure system that was moving slowly from the west to the east of the European continent. Polar, warm maritime air masses were passing over Poland. During the measurement period of 18 September 2018, the sky was almost cloudless, and the wind in the afternoon was weak (below 2 m s^−1^). At night, local radiation fog was observed close to the ground level at flight point. Sunset for the city of Krakow was at 16:50 UTC, and flights were conducted from 15:00 UTC until 21:00 UTC.

**Figure 9:**
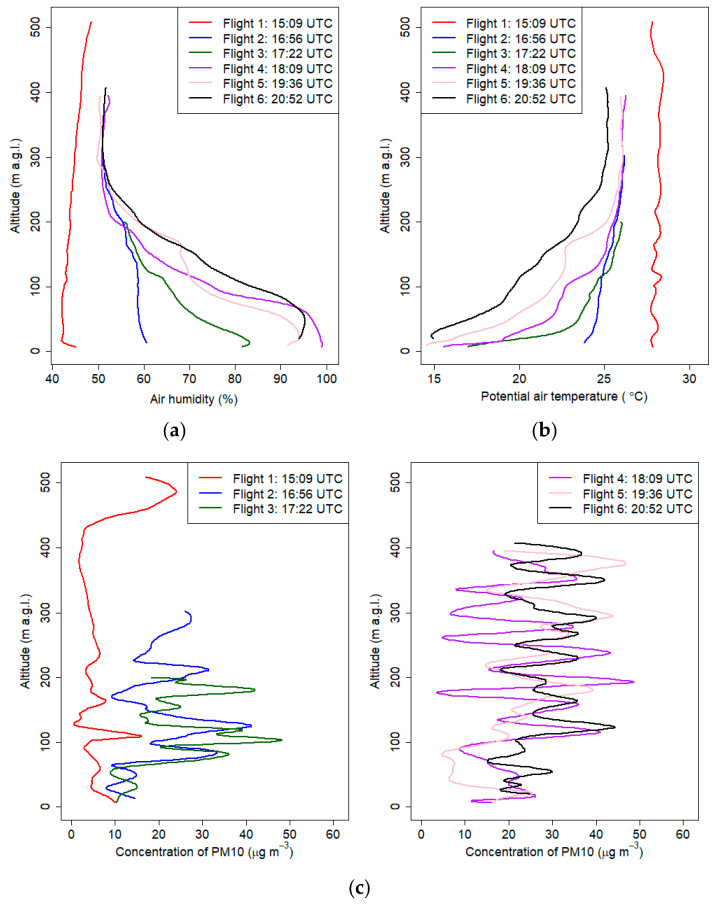
Vertical profiles of relative humidity (**a**), potential air temperature (**b**) and PM_10_ concentration (**c**) on 18 September 2018 from 15:00 UTC to 21:00 UTC.

Figure 9 presents vertical profiles obtained during this campaign. The maximum altitude of the measurements varied between individual flights from 200 m a.g.l. up to 500 m a.g.l.

First measurements were made 2 h 40 min before the sunset. The vertical profile of the potential temperature and relative humidity at the first flight was almost constant, and maximum differences in the potential temperature were approximately 0.8 °C. Starting from the third flight (16:32 UTC), formation of ground thermal inversion was observed. The process started before the sunset, because of strong cooling of the ground at clear sky conditions.

The observed mean gradient of the potential temperature from the ground level up to 300 m a.g.l. at the sixth flight was equal to 3.3 °C/100 m. The largest change in potential air temperature was measured during the third flight. Differences in the potential air temperature between 7 m a.g.l. and 40 m a.g.l. were 6.1 °C (the potential temperature gradient was equal to 18 °C/100 m). Similar phenomena were presented in Banta et al. 2007 [46] defined as a very stable boundary layer and as a shallow boundary layer with a thickness of 10–30 m with weak intermittent turbulence within the strong surface-based radiation inversion. Because of strong surface cooling at night, we observed radiation fog, which was also visible in the vertical profiles of air humidity after 18:00 UTC. Air humidity measurements suggested that the upper limit of the fog was 60 m a.g.l. Subsequent vertical air humidity profiles above 290 m a.g.l. did not differ significantly for flights 2 and 6. Measurements of vertical profiles of potential temperature and relative humidity indicated that in the valley, a cold air pool developed below valley depth. Significant decrease of air humidity and potential temperature in the layer between 60 and 300 m a.g.l. indicates that at the upper layers above the valley, advection of drier and warmer air masses occurred.

The PM_10_ concentration in the vertical profile measured at 15:09 UTC also confirmed that the lower part of the planetary boundary layer (PBL) was homogeneous, and values from the ground up to 400 m a.g.l. were less than 20 µg m^−3^. PM_10_ concentration values observed during the first flight were higher in the upper part of the profile, with the maximum value of ca. 30 µg m^−3^ observed close to maximum altitude. Higher PM_10_ values observed in the upper part could be caused by an emission plume originating from a powerplant located ca. 8.5 km southwest from the flight location.

During the night, the PM_10_ concentration in the profile increased, but the maximum concentration in all profiles did not exceed 50 µg m^−3^. Analysis of vertical profiles of PM_10_ concentration indicates that pollution in the Vistula River Valley in the layer up to 50–70 m a.g.l. did not vary significantly (small fluctuations). However, during flights 2–6, at a layer above 100 m a.g.l. (valley depth), significant fluctuations in pollution were visible. Possible explanation of this phenomenon is occurrence of pollutant transport from distant areas or influence of residual turbulence on pollutants trapped in the ground inversion layer and residual layer located above.

Second possible scenario is occurrence of a nocturnal low-level jet above valley depth, which can modify the dispersion of pollutants. The long-term studies for the period 1994–1999 with the use of SODAR for this region showed the presence of a significant wind direction change during the day in the cold season and in the warm season during the night. The largest wind direction change was observed in the layer of 100–150 m a.g.l. Additionally, attention should be paid to the frequent occurrence of a significant wind speed increase in the layer 50–170 m a.g.l. during the night in the warm season (nocturnal low-level jet) [29].

**Figure 10:**
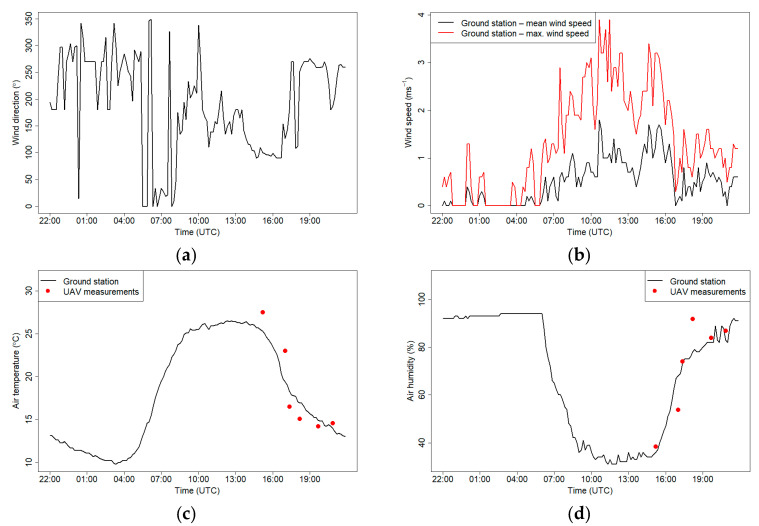
Wind direction (**a**), wind speed (**b**), air temperature (**c**) and relative humidity (**d**) measured at the JU Campus meteorological station on 18 September 2018. Red dots show measurements from the drone that were made at the start of each flight.

Figure 10 presents the air temperature, humidity and wind conditions on 18 September 2018. During the drone measurements, the mean wind speed was below 2 m s^−1^, with a maximum speed reaching 3 m s^−1^; between 14:00 UTC and 16:30 UTC, the wind direction was south, but between 16:30 UTC and 17:30 UTC, the direction changed to west-south-west. Maximum air temperature measured on the JU Campus was 26.4 °C at 13:40 UTC. Air temperature during the first four flights measured by a drone differed from the measurements conducted at the Campus JU station, and the differences ranged from 1.8 ± 0.6 °C to 3.6 ± 0.6 °C. The differences during the afternoon hours may be caused by well-developed thermal turbulence, which generates significant microclimatic variability in the air temperature and relative humidity. The turbulence is the result of the differences in air temperature over various active surfaces which can be found around the measurement site and the campus. Each active surface shows a different structure of the heat balance [47]. Air parcels of various properties are generated over each type of the active surface, and then they are moved freely so the sensors measure the properties of accidental air parcels. During the night, when the thermal turbulence is much weaker, the air temperature measurements from both sources were consistent, and the maximum difference in air temperature in flights 5 and 6 was 0.7 ± 0.6 °C. Measurements of air humidity during almost all the flights did not differ significantly from measurements made at the station, except for measurements during flight 4 when the difference was 15%.

**Figure 11:**
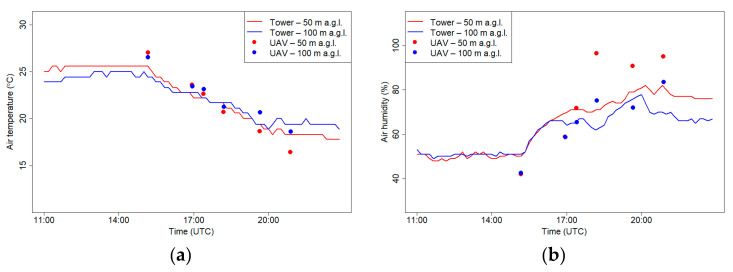
Air temperature (**a**) and relative humidity (**b**) at 50 and 100 m a.g.l. measured at the RTCN tower and by UAV on 18 September 2018.

Figure 11 presents measurements of air humidity and temperature on 18 September 2018 that were measured at the RTCN tower and using a drone at the following two levels: 50 m a.g.l. and 100 m a.g.l. Observations of air temperature and humidity that were conducted using a drone during the day (first flight) were slightly different from the tower site for both levels. Temperatures measured using a drone were approximately 2 °C higher than the tower, and relative humidity was approximately 8% lower, but the observed gradients between 50 and 100 m a.g.l. were the same for both sites. For the later flights, the temperature that was measured using the drone was lower, and the relative humidity was higher than the tower results, especially for 50 m a.g.l. The differences in meteorological conditions between the two places were possibly caused by different surface radiation budgets because of differences in land cover that was adjacent to both sites. The second possibility of significant differences between layer 50 and 100 m a.g.l. is topographic relief; in the west from the RTCN tower are hilltops with altitudes that reach from 300 to 400 m a.s.l. (Figure 7, RTCN tower—point no. 3), while at the nearest region to the flight place, the altitude varies between 200 and 250 m a.s.l. Due to this fact, the tower station may be under the influence of topographic shading at selected atmospheric conditions (i.e., western advection). On the other hand, inconsistences between relative humidity measurements can be partially caused by insufficient response time correction of the sensor on the drone (see Figure 6b).

### 3.2. 21 September 2018—Morning Campaign

On 21 September 2018, Poland was under the influence of a high-pressure system that was located on the border between Ukraine and Moldova. In the morning, the sky was clear, and fog was observed from the ground level to a height of several dozen meters. At the synoptic station of Balice, visibility until 7 UTC did not exceed 10 km; in the valley, atmospheric silence occurred (wind speed below 1 m s^−1^).

**Figure 12:**
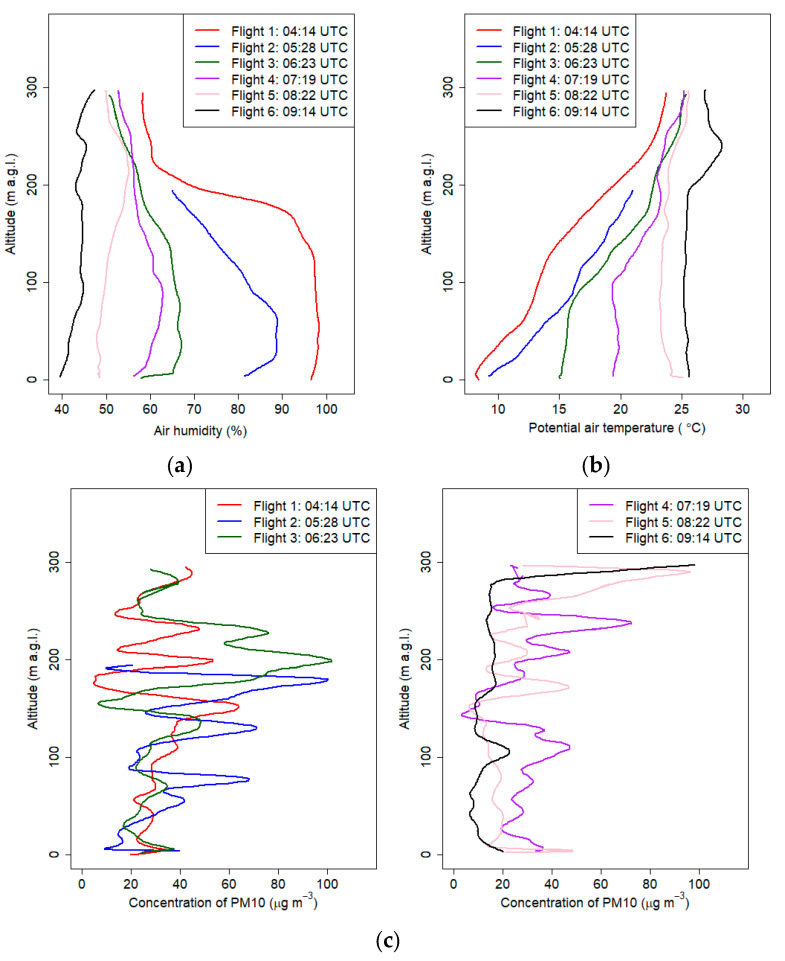
Vertical profiles of relative humidity (**a**), potential air temperature (**b**) and PM_10_ concentration (**c**) on 21 September 2018 from 4:00 UTC to 12:00 UTC.

Figure 12 presents the results of vertical profiles obtained during the flights from September the 21st. The first flight started before sunrise, and profiling was continued until forenoon. Sunrise for that day for Krakow was at 4:22 UTC, and measurements were conducted from 4:00 UTC to 9:30 UTC. Maximum altitude of the measurement ranged from 200 m a.g.l. to 295 m a.g.l.

During the early morning hours, before sunrise, atmospheric conditions were stable (cold air pool developed in the valley), which was confirmed by the vertical profiles of potential air temperature from the first and second flights (the depth of the ground inversion layer at the first and second flights was equal to 230 and 190 m. a.g.l., respectively) (Appendix A, Table A3). The gradient of potential air temperature for both flights was 5.2 °C/100 m (between 0 m a.g.l. and 295 m a.g.l.) and 6 °C/100 m (between 0 m a.g.l. and 195 m a.g.l.), respectively. During subsequent flights, the stable boundary layer gradually disappeared as a result of surface heating. Vertical profiles from flights 3 and 4 indicated the disappearance of the near-ground inversion (neutral conditions in the lowest layer with a depth of ca. 100m a.g.l.) and the appearance of the upper inversion layer. Vertical potential temperature gradient in the layer between 200 and 100 m a.g.l was equal to 5.4 °C/100 m and 3.9 °C/100m during the third and fourth flights, respectively. After sunrise, the air layer close to the ground quickly heated up, which triggered convection processes. Vertical profiles of potential air temperature from flights 5 and 6 (after 8:00 UTC) presented a developed mixed layer with constant potential temperature throughout the profile. Significant differences of the vertical profile of air humidity between the first and second flights at a layer between 70 and 200 m a.g.l. indicate that advection of dry warmer air masses caused destruction of the cold air pool from upper layers.

During the last flight, we observed a thermal plume between 190 and 250 m a.g.l. The maximum difference between the potential temperature in the plume and the surrounding air was 2.8 °C, while the maximum value was measured at 240 m a.g.l.

Accurate analysis of the subsequent pollutant concentration profiles provided valuable information. First flight in the early morning indicated that PM_10_ concentration at the ground layer up to 150 m a.gl. slightly increased with the altitude; at the layer above were visible significant fluctuations of PM_10_ concentration. After the sunrise, vertical profiles of PM_10_ concentration changed because of convective movements.

A significant change in the PM_10_ concentration on the first flight was measured at 150 m a.g.l. Due to the fact that the PM_10_ sensor inlet was heated, the error caused by significant air humidity change was reduced (provided by laboratory experiments not shown in the article). Sudden change of PM_10_ concentration close to the transition layer observed at the vertical profile of air humidity was probably caused by new particle formation (NPF), which was also observed at the top of the atmospheric boundary layer (ABL) in different studies [32]. Research by Platis et al. (2016) [48] indicated that the thermodynamic conditions such as high thermodynamic fluctuations, strong gradients of temperature and humidity near the top of the ABL were favorable for the NPF. Vertical profiles of PM_10_ from the second and third flights showed that at an altitude of ca. 200 m a.g.l. transport of pollutants occurred; in subsequent flights, increased PM_10_ concentration was not visible at this altitude, which is probably caused by a change of meteorological conditions (wind vertical profile and upward convection movements). The PM_10_ concentration in the vertical profile up to 250 m a.gl. was significantly reduced after 9:00 UTC, and the values did not exceed 20 µg m^−3^, except local maximum at 100 m a.g.l. (24 ± 6 µg m^−3^).

**Figure 13:**
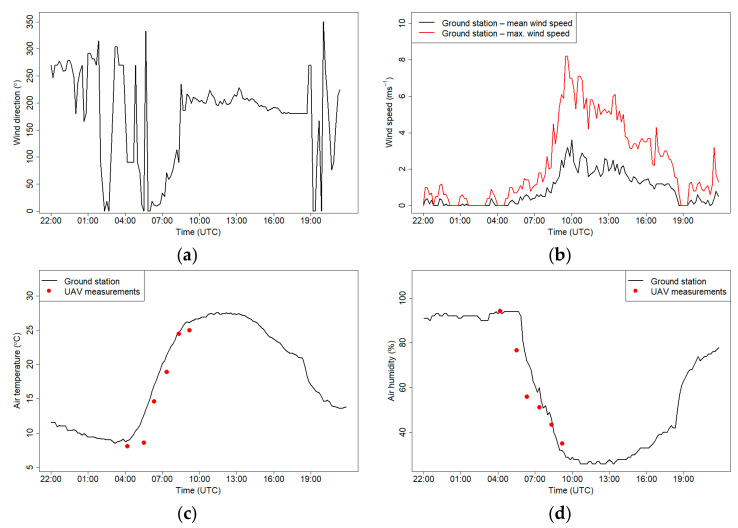
Wind direction (**a**), wind speed (**b**), air temperature (**c**) and relative humidity (**d**) measured at the JU Campus meteorological station on 21 September 2018. Red dots show measurements from the drone made at the start of each flight.

Figure 13 shows meteorological conditions on 21 September 2018. The wind direction during the morning was changing from north to west-southwest at 8:30 UTC, mean wind speed during the night (until 8:00 UTC) was less than 1 m s^−1^, and maximum wind speed during the measurements was 8 m s^−1^ at 10:00 UTC. Measurements of air humidity and temperature that were obtained using the drone were consistent with measurements from the meteorological station.

**Figure 14:**
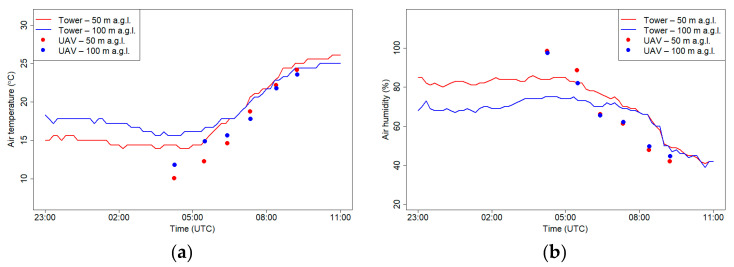
Air temperature (**a**) and relative humidity (**b**) at 50 and 100 m a.g.l. measured at the RTCN tower and by UAV on 21 September 2018.

Figure 14 presents measurements of air humidity and temperature on 21 September 2018 at the RTCN tower and using a drone at two levels 50 m a.g.l. and 100 m a.g.l. Measurements at both levels differed significantly in the first two flights when air temperature from the drone was lower than the observations from the RTCN tower. Vertical profiles of air temperature and air humidity from the tower station indicated that at the western part of the Vistula Valley, during the night, ground inversion occurred. Differences between layer 50 and 100 m a.g.l. in air humidity points out that during the night there was advection of warmer, drier air close to the valley depth. After sunrise, observations from both places did not differ significantly. Lower air humidity at the flight location in comparison with measurements from the tower station after the third may have been caused by ventilation condition differences of both places.

### 3.3. 7 March 2019—Whole-Day Campaign

On 7 March 2019, air advection to Poland from the southwest was caused by the influence of the high-pressure system above Russia and the low-pressure system over the British Isles. The movement of both pressure systems to the east resulted in the inflow of a cold front on that day in the afternoon hours. Measurements from the synoptic station of Balice indicated that high cloudiness during the measurement day maintained up to 14 UTC (6–7 octants), and between 14 and 17 UTC, a sudden decrease in cloud cover up to 2 octants and subsequent increase in cloud cover occurred. Sunrise on that day occurred at 5:12 UTC, until 9 UTC there was a weak wind below 2 m s^−1^, and afterward, there was an increase in wind speed in the Vistula River Valley.

**Figure 15:**
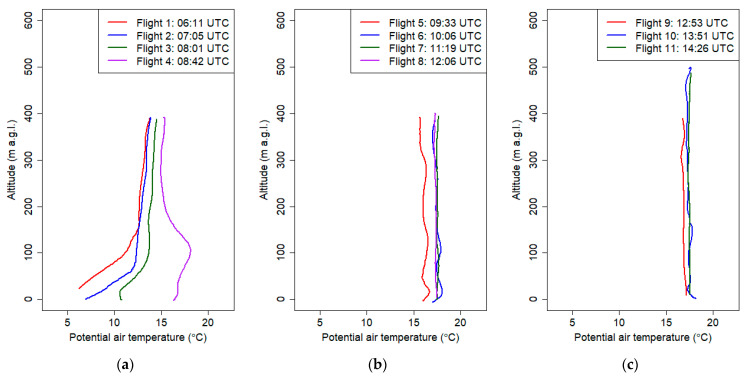
Vertical profiles of potential temperature on 7 March 2019 from 6:00 UTC to 15:00 UTC.

**Figure 16:**
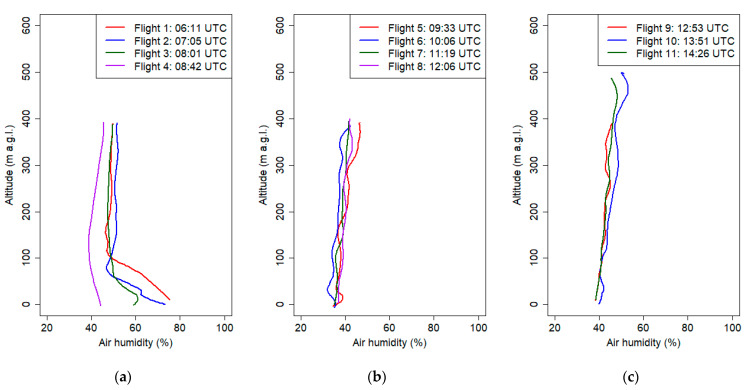
Vertical profiles of relative humidity on 7 March 2019 from 6:00 UTC to 15:00 UTC.

**Figure 17:**
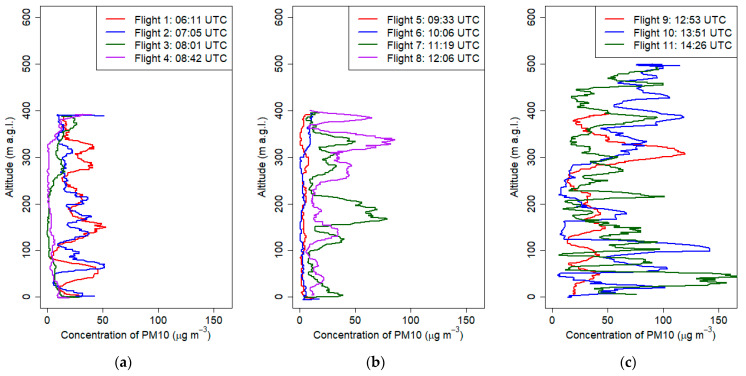
Vertical profiles of PM_10_ concentration on 7 March 2019 from 6:00 UTC to 15:00 UTC.

**Figure 18:**
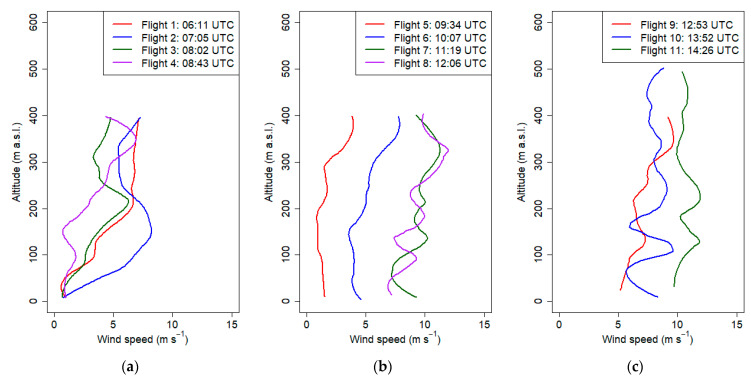
Vertical profiles of wind speed on 7 March 2019 from 6:00 UTC to 15:00 UTC.

The last presented campaign was done on March the 7th 2019 between 6:00 and 15:00 UTC. The aim was to observe the development of foehn wind occurring in the Tatra Mountains (ca. 100km south from Krakow). The first flight (Figure 15) indicated that the thermal inversion reached up to 150 m a.g.l. (potential temperature gradient equal to 5 °C/100 m); during subsequent flights (2–4), its height decreased up to 100 m a.g.l., and the vertical gradient of relative humidity and potential temperature decreased as a result of soil heating and advection of warm air masses associated with the occurrence of the foehn wind. Flights 4-11 indicated that at strong vertical mixing (well-mixed air), vertical gradient of potential temperature and relative humidity during these flights were equal on average to −0.1 °C/100 m and 1.9%/100 m, respectively (Figure 15 and Figure 16). Air humidity in the vertical profile during the measurement campaign did not vary significantly; however, in the first three vertical profiles up to 100 m a.g.l., relative air humidity varied between 40% and 50%. The vertical profiles of PM_10_ for the first two flights (Figure 17a) showed moderate values (up to 50 μg m^−3^) with fine structure including local concentration peaks at 80 m a.g.l. which was probably connected to the change of vertical gradient of relative air humidity from −29%/100 m in the ground layer up to 100 m a.g.l. at first flight and −33%/100 m in the ground layer up to 80 m a.g.l. to almost 0%/100 m at the upper layer. Later flights until 10:00 UTC showed low constant values, which was an effect of foehn wind intrusion into the valley and exchange of air masses (Figure 17a,b). After 11:00 UTC, the PM_10_ concentration started to rise (up to 150 μg m^−3^), and profiles become irregular (Figure 17b,c). One possible explanation is long-range transport of particulate matter by strong foehn wind. The second possibility is that ascending airflow lifted particles from the ground, and therefore at the 10th and 11th flights, high concentration peaks in the layer up to 150 m a.g.l were observed. Final interpretation requires deeper analysis to be performed in another study. The wind speed profile showed the evolution of strong wind gradually penetrating deeper parts of the valley where the city is located. The first five vertical profiles (Figure 18a,b), with the exception of flight 2, indicated that horizontal wind speed in the layer up to 80–100 m a.g.l. did not exceed 2 m s^−1^, which was partially caused by topographic relief (Vistula River Valley depth). Significant increase of wind speed from the ground level up to 150 m a.g.l. (local wind maximum) at second flight was caused by a sudden intrusion of foehn wind from the East into the valley (eastern wind at 7 UTC at the JU Campus station). Sudden temporal intrusion of foehn wind is characteristic of this phenomenon which was confirmed by other studies [49,50].

During the last flight, a strong wind speed exceeding 10 m s^−1^ was observed in the whole profile (Figure 18c).

**Figure 19:**
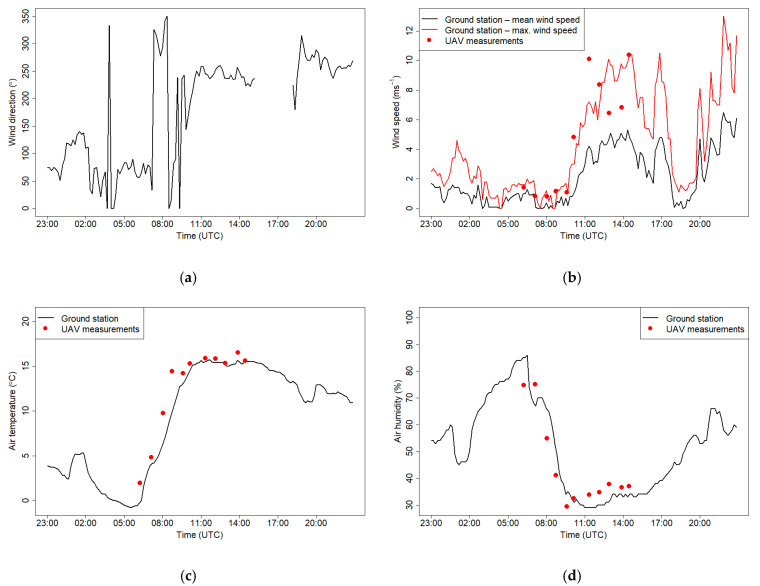
Wind direction (**a**), wind speed (**b**), air temperature (**c**) and relative humidity (**d**) measured at the JU Campus meteorological station on 7 March 2019. Red dots show measurements from the drone made at the start of each flight.

Figure 19 shows meteorological conditions at the JU Campus station on 7 March 2019. The wind direction during the morning was changing from east to west at 9:00 UTC, wind speed during the night (until 9:00 UTC) was less than 1.5 m s^−1^, and maximum wind speed during the measurements reached 10 m s^−1^ at 14:30 0 UTC.

Increase of wind speed being the effect of foehn wind was associated with temperature increase and significant drop of relative humidity between 6 and 9 UTC at the ground station. Measurements of air humidity and temperature that were obtained using the drone were consistent with measurements from the meteorological station. Due to the fact that temporal resolution of the wind component data from the ground meteorological station is the mean values of the 10-min period, Figure 19b presents mean and maximum wind speed measurements. Wind speed data from the sensor located in the UAV have 1 s resolution, therefore during windy conditions, single measurement points can fluctuate significantly. Measurements from the drone conducted at 10 m a.g.l. at the first five flights during weak wind conditions were consistent with observations from the ground station. During the subsequent flights, temporal measurements of wind speed from the drone varied between mean and maximum wind speed observed at the JU ground station for most of the cases, except the sixth and seventh flights.

**Figure 20:**
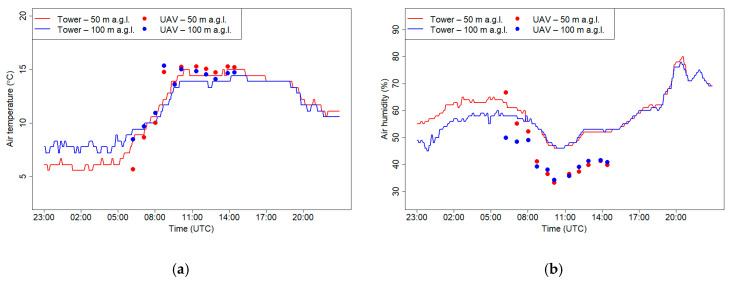
Air temperature (**a**) and relative humidity (**b**) at 50 and 100 m a.g.l. measured at the RTCN tower and by the UAV on 7 March 2019.

Figure 20 presents measurements of air humidity and temperature on 7 March 2019 at the RTCN tower and using a drone at two levels, 50 m a.g.l. and 100 m a.g.l. In the first part of the record, a clear vertical gradient of temperature and relative humidity was observed. The same effect was confirmed by the data from flights. In the later part, after increase of wind speed, a strong turbulence in the atmosphere caused the vertical gradients to disappear. Temperature data from the RTCN and drone were consistent while the relative humidity recorded by the drone during a higher wind speed period was lower by 10% compared to tower observations at both elevations (50 and 100 m a.g.l.).

## 4. Discussion

The results obtained during the presented campaigns using an airborne multisensor measurement platform enabled detailed analysis of temporal variation of vertical PBL structure at a local scale during three different synoptic conditions. Results from morning campaigns (21 September 2018 and 7 March 2019) pointed out that in the layer with strong gradients of temperature and humidity near the top of the ground inversion layer, peaks of PM_10_ concentration were observed. During stable conditions, PM_10_ concentration in the ground layer up to 100 m a.g.l. (valley depth) did not vary significantly, while at the upper layers, strong fluctuations of PM_10_ concentration were observed. Possible explanation is weakening of mechanical turbulence in the valley due to topographic shading and occurrence of nocturnal low-level jet above the valley which can induce mechanical turbulence and modify pollutant dispersion.

Other research presenting the occurrence of a layer with increased concentration of air pollution at a certain height points out that these conditions were connected to vertical diffusion—increased concertation of pollutants at a layer a few dozens of meters above the ground [51]—and diffusion of plumes from elevated sources—upper layers at an altitude of a few hundred meters above the ground [52] or downward flows of regional pollutants from the upper layer [53].

Studies of vertical profiles of potential air temperature and relative humidity pointed out that an important factor that affects cold air pool break is advection of warmer air above the valley during morning hours. At the first campaign (18 September 2018), advection of warmer air strengthened the ground inversion layer during the night. Observed potential temperature gradient up 100 m a.g.l. at sixth flight (after sunset) was equal to 6.3 °C/100 m, while at first flight on 21 September 2018 (before sunrise) was equal to 5.4 °C/100 m.

The temperature data obtained during the first and second campaigns were used for comparison of atmospheric processes occurring during convective and stable nocturnal boundary layer formation. In particular, a potential temperature gradient and the altitude associated with its rapid change were analyzed for subsequent flights. While the first two profiles obtained during the campaign representing the formation of stable nocturnal boundary (15:09 UTC and 16:56 UTC) were almost constant (temperature gradient below 0.3 °C/100 m), next profiles showed temporal increase of nocturnal boundary layer (NBL) depth (defined as the altitude where potential temperature is stabilizing) starting from ca. 100 m a.g.l observed during flight no. 3 and reaching the end value of ca. 300 m a.g.l. for flight 6 (Figure 9b). In the first phase of NBL formation (flight 3), a very steep gradient was observed close to the ground level (ca. + 18 °C/100 m) which gradually disappeared during the next flights when the NBL depth was increasing. The gradient value in the upper part of NBL was almost constant for flights 4–6 (ca. +3.3 °C/100 m). During the second campaign representing the formation of the convective boundary layer, potential temperature profiles looked different. In the initial state before sunrise (first flight at 4:14 UTC), a clear thermal inversion layer reaching the height of ca. 300 m a.g.l. was observed. The potential temperature gradient within the NBL layer was equal to ca. 5 °C/100 m. Starting from flight 3 (6:23 UTC), a formation of the convective layer was observed close to the ground. The surface temperature was increasing for subsequent flights and remained constant in the first part of the profile. The depth of the convective layer (defined as the maximum altitude where the temperature was close to the surface) was increasing with time starting from ca. 80 m a.g.l for flight 3 and ending up with ca. 200 m a.g.l. for flight 6. Above the observed convective layer, a residual layer was still present, and the potential temperature gradient within this layer remained constant.

The comparison of the results of the wind speed from the drone with observations from the JU Campus meteorological station showed good agreement for weak wind conditions (below 2 m s^−1^), while during stronger wind speed occurrence, drone measurements were in between average and maximum wind speed recorded by the JU station, being in most cases closer to the maximum values at a certain moment. Such an effect can be explained by local reduction of wind speed at the JU station location due to the influence of the surrounding campus buildings while the flights were made in the open field area.

Analysis of the period with occurrence of foehn wind in the valley allowed us to identify two opposite effects associated with elevated wind speed. On the one hand, an improvement of the air quality induced by the intense dispersion of pollution stimulated by strong turbulences associated with high wind speed in the first phase of foehn wind (wind speed below 5 m s^−1^) was observed (Figure 17a,b, flights 3–6), but on the other hand, deterioration of air quality by resuspension of deposited particles from the ground was observed in the second phase when wind speed exceeded the value of 5 m s^−1^ (Figure 17b,c, flights 7–11). A similar effect was reported by Kishcha et al. [54] for the Dead Sea Valley region.

An attempt was made to calculate the turbulent kinetic energy (TKE) parameter which can be very useful to identify thermal winds occurrence in the valley; however, drone position fluctuations during the flights in turbulent conditions masked recorded wind components fluctuations required for its calculation, and the obtained values of TKE were extremely noisy and did not show any pattern along the vertical profiles. Future studies are required to test if the analysis of drone fluctuations itself can be useful for determination of the TKE parameter.

## 5. Conclusions

The availability of high-quality atmospheric measurements by using a UAV provides unquestionable value to meteorological studies. However, attention should be paid to the time resolution of the equipment used to measure meteorological (air temperature, wind components, air pressure and air humidity) and air quality parameters (e.g., PM_10_ concertation, amount of particulate matter at selected size). The recommended time resolution of meteorological parameters should be 1s or less [30]. Recent studies indicated that the location of the measuring equipment on the drone is very significant; it can cause two significant problems: bias measurement related to exposure to external radiation sources (e.g., insufficient solar shielding) and heat (e.g., engines or electronics) [30,55]. Position of the sensors on the drone should provide proper ventilation undisturbed by drone propeller movement (quick sensor response to changing environmental conditions).

Several other studies that investigated the effects of a multicopter on air sampling pointed out similar conclusions. The results of computational fluid dynamics (CFD) simulation for the quadrotor with added sampler presented in the article by McKinney et al. 2019 [56] and field studies of the flow of a four-rotor drone using colored pyrotechnical smoke cartridges [57] confirm that the largest airflow disturbances exist at the propellers (especially below the propellers) and the smallest in the middle of the drone above the propeller position. Hence the optimal position of the sensors is in the middle part of a UAV and elevated above the propeller height.

The smaller size of a UAV makes operation much simpler and easier but may reduce the resistance capability of strong winds and increase the instability of measurements significantly [32]. It was confirmed that fluctuations of drone position influence wind speed variability making reliable calculation of, e.g., the TKE parameter, impossible. Additionally, tests of low-cost air pollution sensors indicated influence of air humidity on the measurement of PM_10_ concentration (e.g., sensor Mie pDR-1500 [32]). It was demonstrated that equipping the sensor with a heated inlet solves this problem.

Atmospheric measurements by using a UAV can be applied in high-resolution research, verification of modeling results [58] and as an additional source for data assimilation in the lower troposphere [59]. Recent study indicates that UAVs that are able to accurately measure three-dimensional wind might be used as a cost-effective and flexible addition to measurement masts and LIDAR scans [60].

The atmospheric vertical profile results obtained during three campaigns using an airborne measurement system demonstrate the usefulness of this tool in investigating the dynamics of the lower part of the PBL. The results present three different pictures of the transition phase between the nocturnal boundary layer and the convective layer and influence of the foehn wind on the PBL structure in the city located inside the river valley. Campaign 1 showed the transition from convective to stable stratified atmosphere. Campaign 2 showed vertical atmosphere dynamics during the opposite process. The last campaign was dedicated to studying the influence of foehn wind formation on urban PBL structure.

Concentrations of the PM_10_ fraction in air pollution that are associated with temperature and relative humidity measurements along the vertical profile help to better understand meteorological processes influencing the formation of high PM_10_ concentration. Results of experiments during the night indicate that development of a stable nocturnal boundary layer affected higher PM_10_ concentrations at the lowest part of the boundary layer. After sunrise, the PM_10_ concentration above the ground decreases because of convection that dilutes pollutants in the larger air volume and transports them to the higher levels. The last case showed that, during strong wind periods, a long-distance pollutant transport can significantly influence the air quality.

## Figures and Tables

**Table 1:** System technical specifications.

Parameter	Value
Sampling frequency	1 Hz
Weight	235 g
Temperature operating range	−40 ÷ 85 °C
Temperature resolution	0.01 °C
Temperature noise	0.005 °C
Temperature accuracy	±1.0 °C
Temperature response time	1 s
Pressure operating range	300 ÷ 1100 hPa
Pressure resolution	0.0018 hPa
Pressure noise	0.013 hPa
Pressure accuracy	0.12 hPa
Humidity operating range	0 ÷ 100% RH
Humidity resolution	0.008% RH
Humidity noise	0.02% RH
Humidity accuracy	±3% RH
Humidity response time (0–>90%)	1 s
PM operating range	0 ÷ 1000 µg·m^−3^
PM accuracy	±10 µg·m^−3^
PM single response time	1 s
PM detectable size range, µm	0.3–10
PM size bins range, µm	0.3–0.5, 0.5–1, 1–2.5, 2.5–5, 5–10, >10
PM estimated concentrations	PM_1_, PM_2.5_, PM_10_

**Table 2:** TriSonica^TM^ sonic anemometer technical specifications.

Parameter	Value
Size	9.1 × 9.1 × 5.2 cm
Weight	50 g
Sampling frequency	1 Hz, 2 Hz, 5 Hz, 10 Hz
Wind speed range	0–50 m·s^−1^
Wind speed resolution	0.1 m
Wind speed accuracy	(0–10 m/s) ± 0.1 m/s; (11–30 m/s) ± 1%; (31–50 m/s) ± 2%
Wind direction range	(x/y) 0–360°; (z) ± 30°
Wind direction resolution	1°
Wind direction accuracy	±1°
Pressure range	50–115 kPa
Pressure resolution	0.1 kPa
Pressure accuracy	±1 kPa
Humidity range	0–100% RH
Humidity resolution	0.1%
Humidity accuracy	±3%
Temperature range (derived from speed of sound and humidity)	−40–80 °C (very fast response time)
Temperature resolution	0.1 °C
Temperature accuracy	±2 °C

**Table 3:** Geographical coordinates of measurement points.

No.	Name of Place	Longitude (°E)	Latitude (°N)	Height (m a.s.l. *)
1	Location of vertical measurement (UAV ** place)	19.898	50.026	209
2	JU Campus meteorological station	19.902	50.026	212
3	RTCN tower	19.909	50.051	222
4	AGH UST *** meteorological station	19.912	50.067	220
5	Synoptic station Balice	19.909	50.051	237

*—meters above sea level (m. a.s.l.). **—unmanned aerial vehicle (UAV). ***—AGH University of Science and Technology (AGH UST).

## Data Availability

Not applicable.

## Appendix A

**Table A1:** Time response of humidity and temperature sensors before and after correction procedure.

Humidity Sensor—BOSH						
Order number	Humidity amplitude (%)	Time before correction	The rate of change before correction (%/s.)	Humidity amplitude (%)	Time after correction	The rate of change after correction (%/s.)
Cooling						
1	22	440	0.050	28	150	0.187
2	15	250	0.060	20	100	0.200
**Mean value**	**19**	**345**	**0.055**	**24**	**125**	**0.193**
Heating						
1	17	500	0.034	25	150	0.167
2	17	430	0.040	23	140	0.164
**Mean value**	**17**	**465**	**0.037**	**24**	**145**	**0.165**
**Temperature sensor—thermocouple**						
Order number	Temperature amplitude before correction (°C)	Time (s)	The rate of change (°C/s.)	Temperature amplitude after correction (°C)	Time after correction	The rate of change after correction (°C/s.)
Cooling						
1	6.3	102	0.062	5.8	90	0.064
2	5.5	100	0.055	5.5	80	0.069
**Mean value**	**5.9**	**101**	**0.058**	**5.7**	**85**	**0.067**
Heating						
1	7	120	0.058	6.6	110	0.060
2	6.1	160	0.038	5.9	110	0.054
**Mean value**	**6.55**	**140**	**0.048**	**6.3**	**110**	**0.057**
**Temperature sensor—BOSH**						
Cooling						
1	7.1	409	0.017	7.6	280	0.027
2	5.5	320	0.017	6.2	140	0.044
**Mean value**	**6.3**	**364.5**	**0.017**	**6.9**	**210**	**0.036**
Heating						
1	5.9	440	0.013	6.2	390	0.016
2	5.7	450	0.013	5.9	400	0.015
**Mean value**	**5.8**	**445**	**0.013**	**6.1**	**395**	**0.015**

**Table A2:** Stability of atmosphere on 18 September 2018.

Potential temperature					
Flight order number	Min. temp. (°C) (height a.g.l.)	Max. temp. (°C) (height a.g.l.)	Depth of lowest layer (m)	Potential temperature gradient in lowest layer (°C/100 m)	Stability of atmosphere
1	25 (499)	28 (7)	>500	−0.4	Neutral
2	24 (16)	26 (300)	>300	0.8	Neutral
3	20 (9)	26 (200)	130	4.6	Inversion
4	17 (10)	26 (392)	150	7.2	Inversion
5	15 (10)	26 (386)	200	4.6	Inversion
6	15 (21)	25 (404)	250	3.4	Inversion
Air humidity					
Flight order number	Min. humidity (%) (height a.g.l.)	Max. humidity (%) (height a.g.l.)	Depth of lowest layer (m)	Relative humidity gradient in lowest layer (%/100 m)	Stability of atmosphere
1	50 (499)	41 (7)	>500	1	Neutral
2	60 (16)	50 (300)	>300	−4	Neutral
3	87 (9)	54 (199)	120	−30	Inversion
4	100 (10)	53 (391)	200	−26	Inversion
5	96 (10)	49 (386)	200	−22	Inversion
6	96 (21)	51 (404)	250	−18	Inversion

**Table A3:** Stability of atmosphere on 21 September 2018.

Potential Temperature					
Flight order number	Min. temp. (°C) (height a.g.l.)	Max. temp. (°C) (height a.g.l.)	Depth of lowest layer (m)	Potential temperature gradient in lowest layer (°C/100 m)	Stability of atmosphere
1	6 (0)	23 (293)	230	7.4	Inversion
2	9 (4)	21 (187)	187	6.3	Inversion
3	15 (5)	25 (290)	90	1.9	Inversion
4	20 (4)	25 (297)	100	0.2	Neutral
5	24 (5)	26 (294)	220	0.7	Neutral
6	26 (3)	27 (298)	>300	0.4	Neutral
Air humidity					
Flight order number	Min. humidity (%) (height a.g.l.)	Max. humidity (%) (m a.g.l.)	Depth of lowest layer (m)	Relative humidity gradient in lowest layer (%/100 m)	Stability of atmosphere
1	58 (293)	98 (0)	200	−16.3	Inversion
2	62 (187)	86 (4)	187	−8.9	Inversion
3	48 (290)	65 (5)	290	−3.9	Inversion
4	52 (297)	55 (4)	90	10	Neutral
5	47 (5)	49 (294)	210	3.6	Neutral
6	38 (3)	46 (298)	>300	2.9	Neutral

**Table A4:** Stability of atmosphere on 7 March 2019.

Potential Temperature					
Flight order number	Min. temp. (°C) (height a.g.l.)	Max. temp. (°C) (height a.g.l.)	Depth of lowest layer (m)	Potential temperature gradient in lowest layer (°C/100 m)	Stability of atmosphere
1	6 (24)	14 (389)	150	5.0	Inversion
2	7 (2)	14 (391)	70	7.4	Inversion
3	11 (12)	15 (386)	100	2.9	Inversion
4	15 (285)	18 (105)	100	1.7	Inversion
5	16 (392)	17 (17)	>400	−0.3	Neutral
6	17 (355)	18 (20)	>400	−0.3	Neutral
7	17 (136)	18 (89)	>400	0.0	Neutral
8	17 (331)	17 (13)	>400	−0.1	Neutral
9	17 (307)	17 (12)	>400	−0.1	Neutral
10	17 (456)	18 (2)	>500	−0.2	Neutral
11	17 (268)	18 (488)	>500	0	Neutral
Air humidity					
Flight order number	Min. humidity (%) (height a.g.l.)	Max. humidity (%) (m a.g.l.)	Depth of lowest layer (m)	Relative humidity gradient in lowest layer (%/100 m)	Stability of atmosphere
1	47 (155)	75 (11)	100	−28.8	Inversion
2	47 (82)	73 (2)	80	−32.6	Inversion
3	48 (199)	61 (12)	60	−14.1	Inversion
4	39 (133)	46 (381)	100	−5.0	Inversion
5	34 (2)	47 (372)	>400	3.0	Neutral
6	32 (34)	43 (386)	>400	1.8	Neutral
7	35 (2)	42 (381)	>400	1.6	Neutral
8	37 (9)	43 (347)	>400	1.3	Neutral
9	38 (10)	46 (390)	>400	2.0	Neutral
10	40 (2)	53 (464)	>500	2.3	Neutral
11	39 (12)	48 (448)	>500	1.4	Neutral

## References

[1] (2019). Air Quality in Europe-2019.

[2] Biswas P., Wu C.Y. (2005). 2005 Critical Review: Nanoparticles and the environment. J. Air Waste Manag. Assoc..

[3] Karlsson H.L., Gustafsson J., Cronholm P., Moller L. (2009). Size-dependent toxicity of metal oxide particles-A comparison between nano- and micrometer size. Toxicol. Lett..

[4] Guo S., Hu M., Zamora M.L., Peng J.F., Shang D.J., Zheng J., Du Z.F., Wu Z., Shao M., Zeng L.M. (2014). Elucidating severe urban haze formation in China. Proc. Natl. Acad. Sci. USA.

[5] Renard J.B., Michoud V., Giacomoni J. (2020). Vertical Profiles of Pollution Particle Concentrations in the Boundary Layer above Paris (France) from the Optical Aerosol Counter LOAC Onboard a Touristic Balloon. Sensors.

[6] Wang D.X., Stachlewska I.S., Song X.Q., Heese B., Nemuc A. (2020). Variability of the Boundary Layer over an Urban Continental Site Based on 10 Years of Active Remote Sensing Observations in Warsaw. Remote Sens..

[7] Baron A., Chazette P., Totems J. (2020). Remote sensing of two exceptional winter aerosol pollution events and representativeness of ground-based measurements. Atmos. Chem. Phys..

[8] Wood C.R., Jarvi L., Kouznetsov R.D., Nordbo A., Joffre S., Drebs A., Vihma T., Hirsikko A., Suomi I., Fortelius C. (2013). An Overview of the Urban Boundary Layer Atmosphere Network in Helsinki. Bull. Am. Meteorol. Soc..

[9] Mahrt L. (2014). Stably Stratified Atmospheric Boundary Layers. Annu. Rev. Fluid Mech..

[10] Pardyjak E.R., Fernando H.J.S., Hunt J.C.R., Grachev A.A., Anderson J. (2009). A case study of the development of nocturnal slope flows in a wide open valley and associated air quality implications. Meteorol. Z..

[11] Li X., Xia X., Wang L., Cai R., Zhao L., Feng Z., Ren Q., Zhao K. (2015). The role of foehn in the formation of heavy air pollution events in Urumqi, China. J. Geophys. Res. Atmos..

[12] Wang K., Chen F.E., Au W., Zhao Z.H., Xia Z.L. (2019). Evaluating the feasibility of a personal particle exposure monitor in outdoor and indoor microenvironments in Shanghai, China. Int. J. Environ. Health Res..

[13] Mukherjee A., Stanton L.G., Graham A.R., Roberts P.T. (2017). Assessing the Utility of Low-Cost Particulate Matter Sensors over a 12-Week Period in the Cuyama Valley of California. Sensors.

[14] Kelly K.E., Whitaker J., Petty A., Widmer C., Dybwad A., Sleeth D., Martin R., Butterfield A. (2017). Ambient and laboratory evaluation of a low-cost particulate matter sensor. Environ. Pollut..

[15] Zheng T.S., Bergin M.H., Johnson K.K., Tripathi S.N., Shirodkar S., Landis M.S., Sutaria R., Carlson D.E. (2018). Field evaluation of low-cost particulate matter sensors in high-and low-concentration environments. Atmos. Meas. Tech..

[16] Badura M., Batog P., Drzeniecka-Osiadacz A., Modzel P. Optical particulate matter sensors in PM2.5 measurements in atmospheric air. Proceedings of the 10th Conference on Interdisciplinary Problems in Environmental Protection and Engineering (EKO-DOK).

[17] Schnitzhofer R., Norman M., Wisthaler A., Vergeiner J., Harnisch F., Gohm A., Obleitner F., Fix A., Neininger B., Hansel A. (2009). A multimethodological approach to study the spatial distribution of air pollution in an Alpine valley during wintertime. Atmos. Chem. Phys..

[18] Chemel C., Burns P. (2015). Pollutant Dispersion in a Developing Valley Cold-Air Pool. Bound. Layer Meteorol..

[19] Mamali D., Marinou E., Sciare J., Pikridas M., Kokkalis P., Kottas M., Binietoglou I., Tsekeri A., Keleshis C., Engelmann R. (2018). Vertical profiles of aerosol mass concentration derived by unmanned airborne in situ and remote sensing instruments during dust events. Atmos. Meas. Tech..

[20] Chilinski M.T., Markowicz K.M., Kubicki M. (2018). UAS as a Support for Atmospheric Aerosols Research: Case Study. Pure Appl. Geophys..

[21] Kunz M., Lavric J.V., Gerbig C., Tans P., Neff D., Hummelgard C., Martin H., Rodjegard H., Wrenger B., Heimann M. (2018). COCAP: A carbon dioxide analyser for small unmanned aircraft systems. Atmos. Meas. Tech..

[22] Guimaraes P., Ye J., Batista C., Barbosa R., Ribeiro I., Medeiros A., Souza R., Martin S.T. (2019). Vertical Profiles of Ozone Concentration Collected by an Unmanned Aerial Vehicle and the Mixing of the Nighttime Boundary Layer over an Amazonian Urban Area. Atmosphere.

[23] Brady J.M., Stokes M.D., Bonnardel J., Bertram T.H. (2016). Characterization of a Quadrotor Unmanned Aircraft System for Aerosol-Particle-Concentration Measurements. Environ. Sci. Technol..

[24] Villa T.F., Jayaratne E.R., Gonzalez L.F., Morawska L. (2017). Determination of the vertical profile of particle number concentration adjacent to a motorway using an unmanned aerial vehicle. Environ. Pollut..

[25] Masic A., Pikula B., Bibic D. Mobile Measurements of Particulate Matter Concentrations in Urban Area. Proceedings of the 28th DAAAM International Symposium.

[26] Weber K., Heweling G., Fischer C., Lange M. (2017). The use of an octocopter UAV for the determination of air pollutants—a case study of the traffic induced pollution plume around a river bridge in Duesseldorf, Germany. Int. J. Educ. Learn. Syst..

[27] Babaan J.B., Ballori J.P., Tamondong A.M., Ramos R.V., Ostrea P.M. Estimation of PM 2.5 vertical distribution using customized UAV and mobile sensors in brgy. up campus, diliman, quezon city. Proceedings of the International Conference Geomatic & Geospatial Technology (Ggt 2018): Geospatial and Disaster Risk Management.

[28] Morawska-Horaska M. (1978). Struktura termiczna dolnej części troposfery i jej wpływ na zanieczyszczenie powietrza w Krakowie. Człowiek Sr..

[29] Godłowska J. (2019). Wpływ warunków meteorologicznych na jakość powietrza w krakowie. Badania Porównawcze i Próba Podejścia Modelowego.

[30] Jacob J.D., Chilson P.B., Houston A.L., Smith S.W. (2018). Considerations for Atmospheric Measurements with Small Unmanned Aircraft Systems. Atmosphere.

[31] Nolan P.J., Pinto J., Gonzalez-Rocha J., Jensen A., Vezzi C.N., Bailey S.C.C., de Boer G., Diehl C., Laurence R., Powers C.W. (2018). Coordinated Unmanned Aircraft System (UAS) and Ground-Based Weather Measurements to Predict Lagrangian Coherent Structures (LCSs). Sensors.

[32] Liu C., Huang J.P., Wang Y.W., Tao X.Y., Hu C., Deng L.C., Xu J.P., Xiao H.W., Luo L., Xiao H.Y. (2020). Vertical distribution of PM2.5 and interactions with the atmospheric boundary layer during the development stage of a heavy haze pollution event. Sci. Total Environ..

[33] Local Regression. https://en.wikipedia.org/wiki/Local_regression.

[34] John F., Sanford W. (2018). Nonparametric Regression in R. An Appendix to: An R Companion to Applied Regression.

[35] Joachim R., Pascal B., Muller J.M., Stephanie M. (2009). The Small Unmanned Meteorological Observer SUMO: A new tool for atmospheric boundary layer research. Meteorol. Z..

[36] Kunz M., Lavric J.V., Gasche R., Gerbig C., Grant R.H., Koch F.T., Schumacher M., Wolf B., Zeeman M. (2020). Surface flux estimates derived from UAS-based mole fraction measurements by means of a nocturnal boundary layer budget approach. Atmos. Meas. Tech..

[37] Miloshevich L.M., Paukkunen A., Vömel H., Oltmans S.J. (2004). Development and Validation of a Time-Lag Correction for Vaisala Radiosonde Humidity Measurements. J. Atmos. Ocean. Technol..

[38] Achberger C., Barring L. (1999). Correction of surface air temperature measurements from a mobile platform. Agric. For. Meteorol..

[39] Jonassen M. (2008). The Small Unmanned Meteorological Observer (SUMO): Characterization and Test of a New Measurement System for Atmospheric Boundary Layer Research.

[40] Savitzky A., Golay M.J. (1964). Smoothing and Differentiation of Data by Simplified Least Squares Procedures. Anal. Chem..

[41] Bromba M.U., Ziegler H. (1981). Application hints for Savitzky-Golay digital smoothing filters. Anal. Chem..

[42] Bokwa A. (2009). Miejska wyspa ciepła na tle naturalnego zróżnicowania termicznego obszaru położonego we wklęsłej formie terenu (na przykładzie Krakowa). Prace Geogr..

[43] Trzepacz P., Wiecław-Michniewska J., Brzosko-Sermak A., Kołosin A. (2015). Miasto w Badaniach Geografów.

[44] Bokwa A. (2010). Wieloletnie Zmiany Struktury Mezoklimatu Miasta na Przykładzie Krakowa.

[45] Arya S.P. (2001). Introduction to Micrometeorology.

[46] Banta R.M., Mahrt L., Vickers D., Sun J., Balsley B.B., Pichugina Y.L., Williams E.L. (2007). The very stable boundary layer on nights with weak low-level jets. J. Atmos. Sci..

[47] Oke T.R. (1987). Boundary Layer Climates.

[48] Platis A., Altstadter B., Wehner B., Wildmann N., Lampert A., Hermann M., Birmili W., Bange J. (2016). An Observational Case Study on the Influence of Atmospheric Boundary-Layer Dynamics on New Particle Formation. Bound. Layer Meteorol..

[49] Cetti C., Buzzi B., Sprenger M. (2015). Climatology of Alpine North Foehn.

[50] Drobinski P., Steinacker R., Richner H., Baumann-Stanzer K., Beffrey G., Benech B., Berger H., Chimani B., Dabas A., Dorninger M. (2007). Fohn in the Rhine Valley during MAP: A review of its multiscale dynamics in complex valley geometry. Q. J. R. Meteorol. Soc..

[51] Strbova K., Raclavska H., Bilek J. (2017). Impact of fugitive sources and meteorological parameters on vertical distribution of particulate matter over the industrial agglomeration. J. Environ. Manag..

[52] Xu Y.W., Zhu B., Shi S.S., Huang Y. (2019). Two Inversion Layers and Their Impacts on PM2.5 Concentration over the Yangtze River Delta, China. J. Appl. Meteorol. Climatol..

[53] Han S.Q., Hao T.Y., Zhang Y.F., Liu J.L., Li P.Y., Cai Z.Y., Zhang M., Wang Q.L., Zhang H. (2018). Vertical observation and analysis on rapid formation and evolutionary mechanisms of a prolonged haze episode over central-eastern China. Sci. Total Environ..

[54] Kishcha P., Starobinets B., Alpert P. Modeling of Foehn-Induced Extreme Local Dust Pollution in the Dead Sea Valley. Proceedings of the 35th International Technical Meeting on Air Pollution Modelling and its Application (ITM).

[55] Greene B.R., Segales A.R., Bell T.M., Pillar-Little E.A., Chilson P.B. (2019). Environmental and Sensor Integration Influences on Temperature Measurements by Rotary-Wing Unmanned Aircraft Systems. Sensors.

[56] McKinney K.A., Wang D., Ye J.H., de Fouchier J.B., Guimaraes P.C., Batista C.E., Souza R.A.F., Alves E.G., Gu D., Guenther A.B. (2019). A sampler for atmospheric volatile organic compounds by copter unmanned aerial vehicles. Atmos. Meas. Tech..

[57] Crazzolara C., Ebner M., Platis A., Miranda T., Bange J., Junginger A. (2019). A new multicopter-based unmanned aerial system for pollen and spores collection in the atmospheric boundary layer. Atmos. Meas. Tech..

[58] Lee S.H., Kwak K.H. (2020). Assessing 3-D Spatial Extent of Near-Road Air Pollution around a Signalized Intersection Using Drone Monitoring and WRF-CFD Modeling. Int. J. Environ. Res. Public Health.

[59] Leuenberger D., Haefele A., Omanovic N., Fengler M., Martucci G., Calpini B., Fuhrer O., Rossa A. (2020). Improving High-Impact Numerical Weather Prediction with Lidar and Drone Observations. Bull. Am. Meteorol. Soc..

[60] Thielicke W., Hübert W., Müller U., Eggert M., Wilhelm P. (2020). Towards accurate and practical drone-based wind measurements with an ultrasonic anemometer. Atmos. Meas. Tech. Discuss..

